# Mitochondria Express Functional Signaling Ligand-Binding Receptors that Regulate their Biological Responses – the Novel Role of Mitochondria as Stress-Response Sentinels

**DOI:** 10.1007/s12015-025-10847-2

**Published:** 2025-01-31

**Authors:** Katarzyna Brzezniakiewicz-Janus, Justyna Jarczak, Adrian Konopko, Janina Ratajczak, Magdalena Kucia, Mariusz Z. Ratajczak

**Affiliations:** 1 Department of Hematology, University of Zielona Gora, Multi-Specialist Hospital Gorzow Wlkp, Gorzow, Poland; 2 Department of Regenerative Medicine, Warsaw Medical University, Warsaw, Poland; 3 Stem Cell Institute at Graham Brown Cancer Center, University of Louisville, 500 S. Floyd Street, Rm. 107, Louisville, Kentucky 40202 USA; 4 Center for Preclinical Studies and Technology, Laboratory of Regenerative Medicine, Medical University of Warsaw, Warsaw, Poland

**Keywords:** Mitochondrial receptors, Complement, Complosome, C3aR, C5aR, Purinergic signaling, P2X7, Nlrp3 inflammasome, ROS

## Abstract

Evidence accumulated mitochondria, as the “powerplants of the cell,” express several functional receptors for external ligands that modify their function and regulate cell biology. This review sheds new light on the role of these organelles in sensing external stimuli to facilitate energy production for cellular needs. This is possible because mitochondria express some receptors on their membranes that are responsible for their autonomous responses. This is not surprising given the widely accepted hypothesis that these intracellular organelles originated from prokaryotic ancestors that fused with eukaryotic cells during early evolution. It has been reported that mitochondria express functional estrogen, androgen, glucocorticoid, 5-hydroxytryptamine, melatonin, and cannabinoid receptors. What is intriguing is recent evidence showing that mitochondria could also be directly regulated by active mediators of intracellular complement (complosome) and intrinsic mediators of purinergic signaling. Accordingly, they express receptors for intracellular complement cleavage fragments (C5a and C3a) as well as for adenosine triphosphate (ATP), which, besides its crucial role in transferring energy in the cells, is also an important signaling molecule interacting with P2X7 receptor expressed not only on the cell surface but also on the mitochondria membrane. Based on this, intrinsic complosome and purinergic signaling mediators emerge as important cooperating regulators of reactive oxygen species (ROS) release from mitochondria and activators of intracellular pattern recognition receptor Nlrp3 inflammasome. This activation within the beneficial “hormetic zone response” regulates cell metabolism, proliferation, migration, and adaptation to the surrounding challenges of the microenvironment in a favorable way.

**Graphical Abstract:**

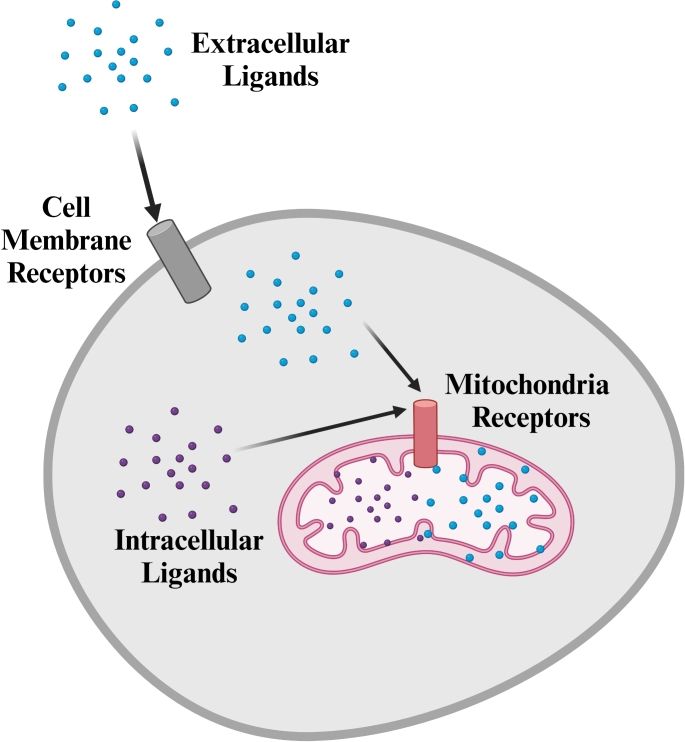

## Introduction

Mitochondria have been reported to express functional receptors for estrogen, androgen, glucocorticoids, 5-hydroxytryptamine, thyroid hormones, melatonin, and cannabinoids. [1–8]. Moreover, recent evidence demonstrated that mitochondria could be directly regulated by mediators of i) intracellular complement (complosome) such as C3a and C5a [9–14] and ii) purinergic signaling mediator nucleotide adenosine triphosphate (ATP) [15]. This sheds new light on these intracellular organelles and their autonomous responses.

Mitochondria are found in the cytoplasm of most eucaryotic cells in the animal, plant, and fungi world. They are called a “powerhouse of the cells” because, they generate ATP through aerobic respiration, serving as a primary energy source for cellular functions [1, 16, 17]. In addition to energy transfer, ATP as a ligand may activate cell surface and mitochondria membrane-expressed receptors and is an essential mediator of the purinergic signaling network. The mitochondria numbers vary with the type of the cells, and hepatocytes have up to 2000 of these organelles per single cell. An exception is red blood cells that remove mitochondria from the cytosol at the terminal stages of erythropoiesis.

Mitochondria are double-membraned, rod-shaped structures ranging from 0.5 to 1.0$\mu$M in diameter and comprise a few compartments carrying out specialized functions. Their electron microscopy structure reveals an outer membrane, an inner membrane, and a gel-like material between membranes called the matrix [16]. Mitochondria possess their own DNA that is similar to bacterial genomes. This fact raised the concept that they are developmental results of fusion between prokaryotic ancestors and eukaryotic cells to ensure aerobic respiration and generate intracellular energy [16, 17]. The composition and number of mitochondrial proteins transcribed from mitochondrial DNA vary depending on the tissue and the species and are dynamically regulated. In the myocardium, characterized by a high oxidative state, mitochondria occupy up to 40% of the cell volume.

Besides their most crucial function, producing energy through aerobic phosphorylation, they are involved in multiple other cellular processes. This includes generating reactive oxygen species (ROS), important in oxidizing cysteine and methionine residues in proteins involved in signal transduction, metabolism, and gene expression [1, 16, 17]. They also contribute to maintaining an adequate concentration of calcium ions within the compartments of the cell, are involved in steroid hormones and heme synthesis, development and function of immune cells, regulation of apoptosis, and reveal pending tissue some specific functions such as in liver cells detoxification of ammonia [1–8]. Because mitochondria DNA is relatively short, several transcription factors in these organelles and receptors are synthesized in the cytosol and transferred to mitochondria.

This short review will present a recent perspective on mitochondria as organelles responsive to external ligands that stimulate and regulate several biological processes in the cell. The results of mitochondrial activation may follow a biological principle of hormesis, which explains how cells adapt to moderate stress to improve their tolerance to more severe stress challenges [18, 19]. Hormesis is characterized by a beneficial effect at low doses of potential stressors and an inhibitory or toxic effect at high doses [18, 19].

## Mitochondria Receptors are Encoded by Nuclear DNA and Transferred to these Organelles after Synthesis in the Cytosol or Acquired by Endocytosis from the Cell Surface Membranes

Mitochondria are autonomous or semi-autonomous organelles because they contain DNA and synthesize about 10% of their proteins [16, 17]. As mentioned above, this indicates that most of the mitochondrial proteins are encoded by cell-nuclear DNA. After translation on ribosomes, these proteins have to be imported into mitochondria. The nuclear DNA encoded mitochondria proteins synthesized on ribosomes in the cytosol, including selected receptors, contain signaling sequences at their N-termini or within their amino acid sequences that allow them to target and enter mitochondria. Moreover, some of the receptors may be transferred from the cell surface membranes by endocytosis. Mitochondria contain two membranes that differ in permeability. The outer mitochondrial membrane is highly permeable and non-selective. In contrast, the inner mitochondrial membrane has limited permeability [16, 17].

The human mitochondrial genome is a circular DNA molecule of about 16 thousand base pairs that encode 37 genes. From these 37 genes, 13 are partially responsible for the subunits of respiratory complexes: I (NADH-dehydrogenase), II (succinate dehydrogenase), III (cytochrome c), IV (cytochrome oxidase), and V (ATP synthase) [1, 16, 17]. However, these subunits are encoded mainly by nuclear DNA and, after cytoplasmatic translation, are imported into mitochondria, where they become assembled into functional complexes with some mitochondrial DNA-encoded proteins. Other 22 genes encode mitochondrial transfer RNAs, and two encode ribosomal RNA [1].

**Fig. 1:**
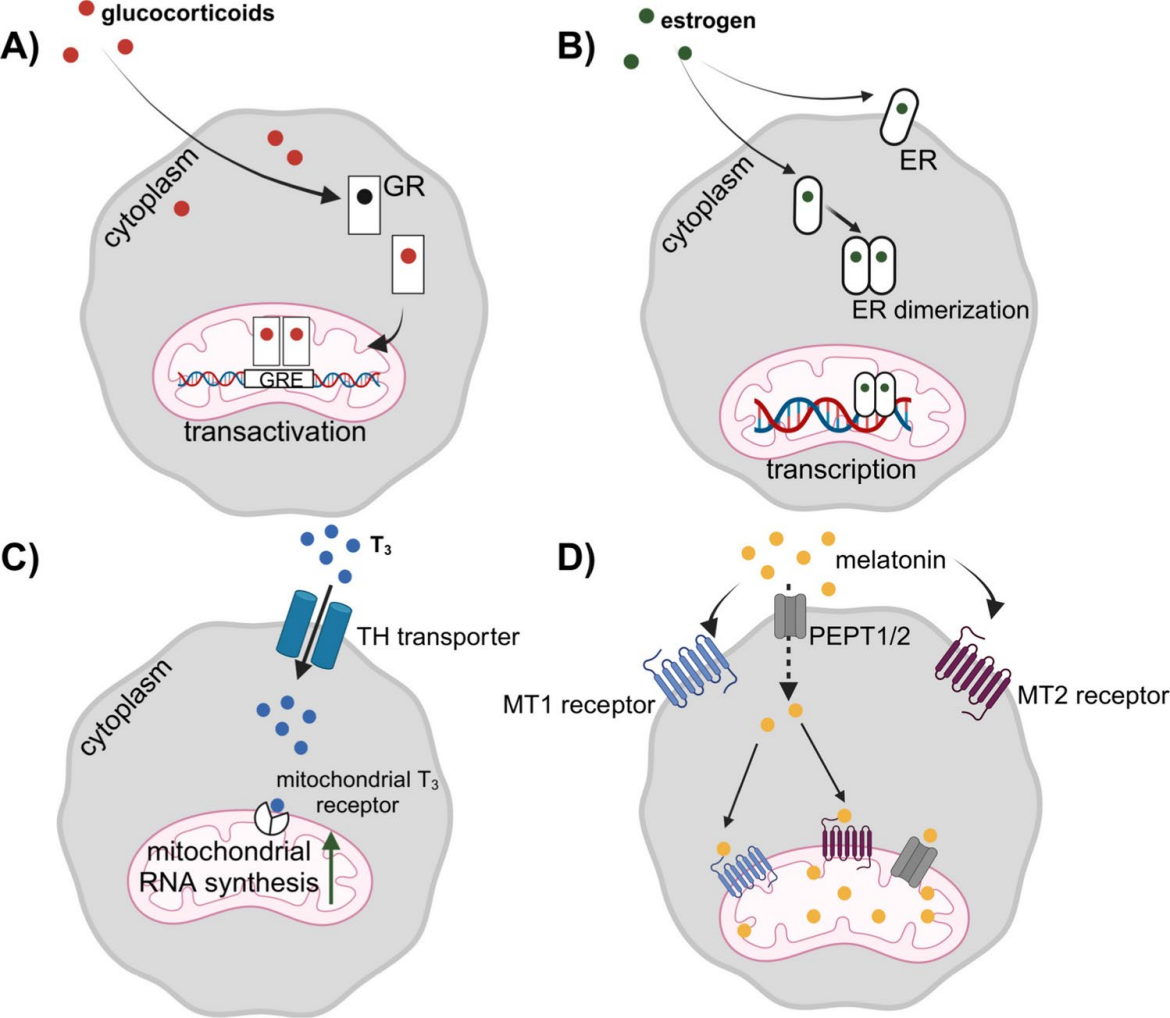
Biological effects of glucocorticoids (**A**), estrogens (**B**), T3 thyroid hormone (**C**), and melatonin (**D**) on mitochondria. Mitochondria express receptors for these hormones, which are transferred from the cell membrane or freshly synthesized in the cytosol. The biological effects of receptor activation include the transactivation of gene expression by mitochondrial DNA, an increase in gene transcription, an increase in mitochondrial RNA synthesis, and the inhibition of stress-related apoptosis

**Fig. 2:**
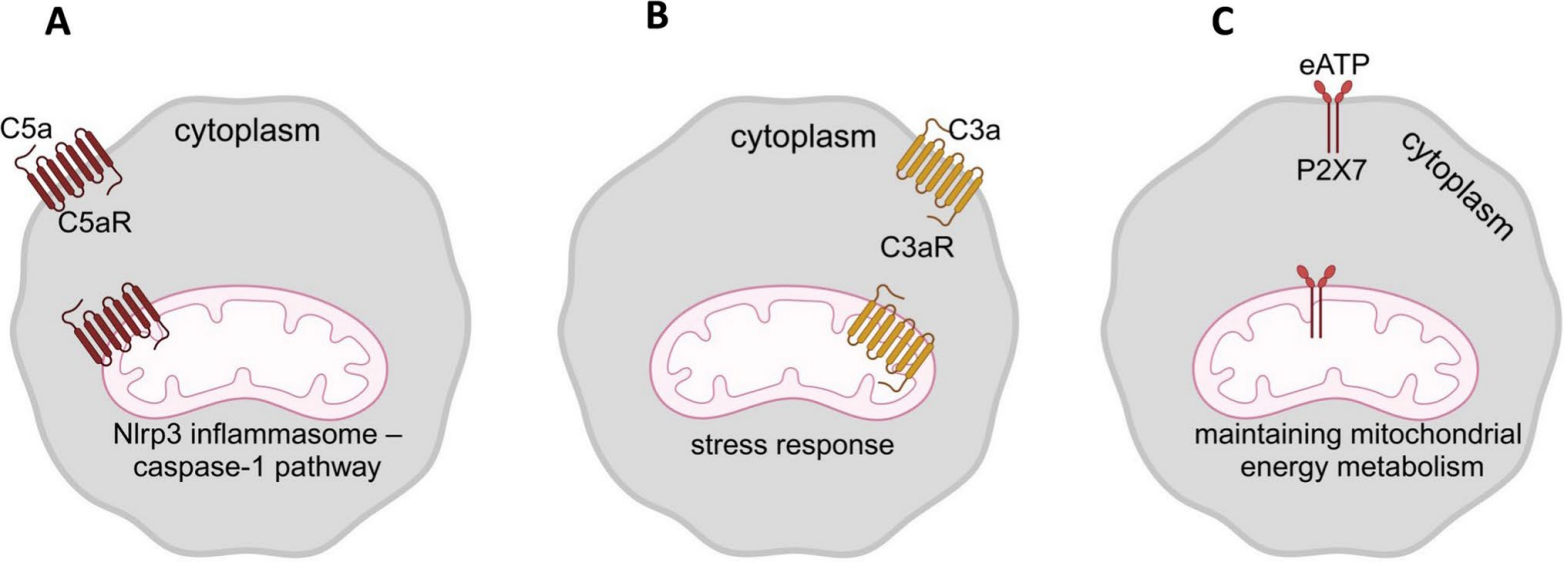
Biological effects of complement C5aR (**A**) and C3aR (**B**) and purinergic receptor P2X7 (**C**) signaling in mitochondria. These biological effects include activating the ROS-Nlrp3 inflammasome pathway, regulating stress response, and maintaining mitochondrial integrity and metabolism

Based on those above, several receptors and transcription factors accumulate in mitochondria after transfer from the cytosol. The most critical receptors that accumulate in mitochondria include i) two estrogen receptors ER $\alpha$ and ER $\beta$, ii) androgen receptor (AR), iii) glucocorticoid receptor (GR), iv) triiodothyronine (T3) receptor, v) 5-hydroxytryptamine 3 (5-HT_3_) receptor, vi) melatonin MT1/2, and vii) cannabinoid (CB1R) receptors [1–8] (Fig. 1). What is very exciting is that mitochondria also express receptors for complement cleavage fragments C3a and C5a (C3aR and C5aR) [9–14], as well as P2X7 purinergic receptor for signaling ATP [15] (Fig. 2). We can expect that the list of mitochondrial receptors is not final and will expand shortly when new receptors are identified on these organelles by employing more sensitive detection techniques. Like the receptors, several transcription factors that operate on the transcription of mitochondrial DNA are also transferred from the cytosol to these organelles.

## Biological Significance of Receptor Expression by Mitochondria

The expression of receptors on mitochondria and their activation leads to several biological effects. Below, we will address the most critical receptor-mediated effects on mitochondria that orchestrate the proper functioning of these organelles. In a recent elegant review [20], it has been proposed that mitochondria function as the processors of the cell, and together with the nucleus and other organelles, they form the “mitochondrial information processing system." Receptors expressed by mitochondria play an important role in this system. It has been suggested that these organelles sense and respond to both endogenous and environmental signals, integrate incoming information through dynamic, network-based physical interactions, and, based on this, adjust the functions of other cell organelles to regulate and coordinate cell physiology [20].

### Estrogen Receptors ER-$\alpha$ and ER-$\beta$

Since estrogen is a steroidal hormone, due to its lipophilic nature, it quickly diffuses through cell membranes [1, 2]. ER-$\alpha$ and ER-$\beta$ receptors are found not only on a cell surface membrane but also in cytosol, nucleus, and mitochondria. Sex-based differences in human disease are partly caused by the levels of endogenous sex steroid hormones (estrogens and androgens), which regulate mitochondrial metabolism [21]. Both membrane-bound and intracellular estrogen receptors mediate different responses after binding to estrogen. While cell membrane-expressed receptors initiate signaling pathways, those expressed in cell nucleus or mitochondria serve mainly as a nuclear and mitochondrial DNA transcription factor, respectively. In mitochondria, their biological effects result in the transcription of some genes and replication of the mitochondrial genome. Estradiol regulates mitochondrial metabolism and morphology through nuclear and mitochondrial-mediated events, including the transcription of nuclear respiratory factor-1 (NRF-1). This factor interacts with coactivators, such as peroxisome proliferator-activated receptor gamma coactivator one alpha (PGC-1$\alpha$) and mitochondrial transcription factor A (TFAM), to control the expression of nuclear-encoded mitochondrial genes. Other estrogen-regulated mitochondrial activities encompass the regulation of the oxygen consumption rate (OCR) and extracellular acidification (ECAR) [21]. It is postulated that ER-$\beta$ serves as the primary estrogen receptor in mitochondria. The overall impact of estrogen signaling results in mitochondrial biogenesis and antioxidant effects [1, 2]. In addition to estrogens, mitochondria can detect and sense androgenic hormones. Research has demonstrated that prostate cancer cells and sperm cells express canonical androgen receptors in mitochondria [20].

### Glucocorticoid Receptor (GR)

The discovery of Glucocorticoid Response Elements (GREs) in the mitochondrial DNA sequences and the physical presence of GRs in mitochondria indicates that these receptors directly regulate gene expression in these organelles [3]. The evidence accumulated that glucocorticoid hormones promote oxidative phosphorylation and mitochondrial energy metabolism. Moreover, mitochondrial GRs modulate apoptosis-mediated processes by interacting with or altering the distribution of Bcl2 family members. It has also been postulated that GR chaperones (Hsp70/90, Bag-1, FKBP51), the anti-apoptotic protein Bcl-2, and the HDAC6- mediated deacetylation and the outer mitochondrial translocation complexes co-ordinate GR accumulation in mitochondria [1, 3].

### Triiodothyronine (T3) Receptor

Mitochondria produce heat primarily through a process called "uncoupling of aerobic phosphorylation" [1, 16, 17]. This allows the body to generate warmth by essentially "wasting" energy as heat instead of fully converting it to ATP. Thyroid hormones significantly regulate this process. When thyroid hormone levels are high, more heat is produced due to increased mitochondrial activity and the activation of uncoupling proteins, mainly in brown adipose tissue. Moreover, triiodothyronine (T3) receptor variant c-ErbA alpha-1 is a major regulator of mitochondrial activity and mitochondrial RNA synthesis [5]. T3 increases the expression of mitochondrial transcription factors mtTFA, mtTFB1, and mtTFB2 required to replicate mitochondrial DNA (mtDNA). This regulates the number of mtDNA copies, ensuring simultaneous coordination of the expression of the mitochondrial genome and nuclear genes encoding mitochondrial proteins. Moreover, T3 is involved not only in the regulation of cellular fuel metabolism but also in the regulation of cell differentiation. T3 receptor-mediated coordination of metabolism and differentiation regulates development [1, 5].

### 5-hydroxytryptamine 3 (5-HT_3_) Receptor

The 5-HT_3_ receptor has five subunits in humans, from A to E. These receptors are expressed on the inner membrane of the mitochondria. The presence of the receptors on the mitochondria membrane changes the membrane potential and mitochondrial oxygen consumption in response to serotonin [4].

### Melatonin MT1/2 Receptors

Melatonin, as an amphiphilic pleiotropic indoleamine, is an ancient molecule with direct and/or receptor-mediated effects. Being an antioxidant and free radical scavenger, it accumulates in mitochondria where it can interact with mitochondria MT1/2 receptors [6, 7]. Melatonin activates NRF2, which is involved in antioxidant defenses and inflammation. It has been demonstrated that by activating the MT1 receptor on the outer mitochondria membrane, melatonin prevents apoptosis induced by hydrogen peroxide in retinal pigment epithelial cells. It also inhibits stress-related release of proapoptotic cytochrome C. Melatonin also stimulates superoxide dismutase 2 (SOD2), a major antioxidant enzyme in mitochondria. All these effects of melatonin direct or MT1/2 receptor-mediated support mitochondrial protective effects of melatonin [1, 6, 7].

### Cannabinoid CB1 Receptor

It has been demonstrated that cannabinoid intoxication leads to amnesia both in humans and animals, and this effect is mediated by mitochondria-expressed CB1 receptors that impair energetic activity in neural hippocampus cells [8]. CB1 receptors in mitochondria signal to engage intra-mitochondrial G$\alpha$_i_ protein activation to inhibit soluble-adenylyl cyclase (sAC). This results in the inhibition of protein kinase A (PKA)-dependent phosphorylation of some mitochondrial electron transport system subunits, and decreased cellular respiration, proving evidence that CB1 receptors in mitochondria regulate memory processes via modulation of energy metabolism [1, 8].

## The Identification of Complosome and Purinergic Signaling Receptors on Mitochondria Opens a New Area of Investigation into the Role of these Organelles in Adaptation to Stress

Mitochondria, because of their pleiotropic functions, were called different names. Based on ATP supply as the “powerhouse of the cell” or due to taking nutrients, breaking them down, and creating energy-rich molecules as a “microscopic digestive system.” Based on a recent discovery that mitochondria express signaling receptors C3aR and C5aR for the complement cleavage fragments C3a and C5a [9–14] and P2X7 purinergic receptors for ATP [15], they could be envisioned and called “stress responsive sentinels.” [21, 22].

To explain this postulated description of mitochondria, the most existential task of the cells is to survive by ensuring proper metabolism, avoiding harmful stimuli, and adapting to changing environments. This requires an energy supply and a balanced redox state, which explains why early evolutionary primordial pathways remained in developing organisms active to regulate cell and tissue integrity [9–14]. Accordingly, the universal intracellular energy transporter purine nucleotide-adenosine triphosphate (ATP) generated mainly during aerobic glycolysis in mitochondria became a vital signaling molecule and precursor of purinergic signaling after being released as a ligand from the cells. Similarly, ancient proteins involved in intracellular metabolism gave rise to the third protein component (C3) of the complement cascade (ComC) during evolution. Both these regulatory pathways induce cytosol reactive oxygen (ROS) and reactive nitrogen species (RNS) that regulate the redox state of the cells. While low levels of ROS and RNS within the beneficial “hormetic phase” [18, 19] promote cell growth and differentiation, supra-physiological concentrations can lead to cell damage by pyroptosis. This balance explains the impact of purinergic signaling and innate immunity on cell metabolism, organogenesis, and tissue development. Moreover, recent evidence shows that both these regulatory pathways operate in a paracrine manner and inside cells at the autocrine level [9–15, 22–24].

To support this, functional receptors for the ComC cleavage fragments C3a and C5a anaphylatoxins, C3aR and C5aR, respectively, and the purinergic receptor P2X7 for signaling ATP have been detected in addition to the cell surface membranes, intracellular in mitochondria [14, 15]. These intriguing observations shed new light on the biological responses of these organelles to stress.

### C5aR and C3aR

These two important complement cascade G-protein coupled receptors were studied for years as cell surface membrane-expressed receptors responding to liver-derived circulating in peripheral blood complement cleavage fragments. The breaking groundwork of Kemper et al. demonstrated that these receptors are also expressed in mitochondria [9–14]. This group reported that mitochondrial C5aR1 in macrophages controls interleukin 1$\beta$ (IL-1$\beta$) production underlying sterile inflammation [14]. This indicates that activation of C5aR in mitochondria leads to the release of ROS that triggers activation of the Nlrp3 inflammasome – caspase-1 pathway [25–27]. It is well known that caspase-1 converts pro-interleukin -1$\beta$ to the mature form released from the cells. Based on this, the mitochondrial ROS-Nlrp3 inflammasome axis is involved in downstream complosome signaling. Moreover, since mRNA for another C5a receptor, C5aR2, has been identified in the cells as part of complosome expression, it would be interesting to investigate its role in intracellular modulating of C5a signaling.

The role of mitochondrial C3aR activation was studied in retina pigment epithelium cells. Interestingly, the cell membrane expressed C3aR in response to oxidative stress was translocated via endocytosis and Rab7-dependent endosomal cargo transfer to mitochondria [12]. Seahorse analysis revealed that activation of C3aR on mitochondria inhibited state II ADP-driven aerobic respiration, which inhibited maximal respiratory capacity. This interesting report demonstrated the importance of balancing extra- and intracellular complement signaling in response to stress [12].

This involvement of mitochondria-expressed C5aR and C3aR in response to stress was confirmed in our recent studies performed with complosome-deficient murine bone marrow (BM) cells. While lineage-negative BM cells from C5-KO cells are more resistant to oxidative stress, the same cells from C3-KO mutants were more sensitive [24]. To explain this, while in C5-KO cells, the biological effects of C3a complosome that is intact prevail, in C3-KO cells, the C5a element of complosome activation dominates. These sensitivity effects to stress are mediated, as we postulate, at the mitochondrial level in complosome-deficient cells [24].

### P2X7 Purinergic Receptor

The role of this receptor has been intensively investigated in murine cardiomyocytes. In an interesting study, it has been reported lack of the P2X7 receptor on mitochondria leads to several dysfunctions of these organelles, including decreases i) in basal respiratory rate, ii) ATP-coupled respiration, iii) maximal uncoupled respiration, iv) resting mitochondrial potential, and v) mitochondrial matrix calcium level [15]. Moreover, the lack of P2X7 changed the expression pattern of some oxidative phosphorylation enzymes and affected heart performance. At the morphological level, hearts from P2X7-receptor cardiomyocytes were enlarged, and mitochondria in cardiomyocytes were smaller than in normal littermates’ hearts. These changes in the metabolism and morphology of cardiomyocytes resulted in decreased stroke volume, ejection fraction, fractional shortening, and cardiac output [15]. In conclusion, the lack of P2X7 receptors on mitochondria from P2X7-KO cells demonstrated the critical role of this receptor as a modulator of mitochondrial energy metabolism. Further studies are needed to investigate the role of mitochondria-expressed P2X7 receptors in addition to cardiomyocytes in other types of cells and organs. It is also important to ask if, besides P2X7, other purinergic receptors from the P2 and P1 receptor family are expressed in these organelles [28].

## Conclusions and Call for Further Research

The mitochondria can not be regarded today as simple powerhouses of the cells. They are living, dynamic, signaling organelles that actively transduce and process biological information. Recent evidence demonstrated also that they can be transferred from cell to cell [29]. Intriguingly, the expression of typical cell-surface receptors on mitochondria opens an exciting area of investigation for their pleiotropic effects. Several receptors have already been identified; we expect more to be determined soon. These receptors could be transferred from the cell surface to mitochondria by endocytosis or are synthesized in the cytosol and moved after assembly to the mitochondria. Mitochondrial receptors could have distinct roles from those expressed on the cell surface, as they directly target intramitochondrial pathways. Some of these receptors promote aerobic glycolysis and reactive oxygen species (ROS) generation, while others exert opposing effects. The expression of complosome receptors (C5aR1, C5aR2, and C3aR) and purinergic signaling receptor P2X7 support the role of mitochondria as intracellular “stress sensing sentinels”. It justifies more research on highlighting the role of mitochondria receptors in different types of cells and developing strategies to modulate the activity of these receptors in several clinical settings.

## Data Availability

This paper does not contain any experimental data.

## References

[1] Lee, J., Sharma, S., Kim, J., Ferrante, R. J., & Ryu, H. (2008). Mitochondrial nuclear receptors and transcription factors: Who’s minding the cell? *Journal of Neuroscience Research,**86*(5), 961–971. 10.1002/jnr.21564 18041090 10.1002/jnr.21564 PMC2670446

[2] Ventura-Clapier, R., Piquereau, J., Veksler, V., & Garnier, A. (2019). Estrogens, estrogen receptors effects on cardiac and skeletal muscle mitochondria. *Frontiers in Endocrinology (Lausanne).,**10*, 557. 10.3389/fendo.2019.00557 10.3389/fendo.2019.00557 PMC6702264 31474941

[3] Kokkinopoulou, I., & Moutsatsou, P. (2021). Mitochondrial glucocorticoid receptors and their actions. *International Journal of Molecular Sciences,**22*(11). 10.3390/ijms22116054 10.3390/ijms22116054 PMC8200016 34205227

[4] Rao, S., Turek, I., Ratcliffe, J., Beckham, S., Cianciarulo, C., & Adi,l S., et al. (2023). 5-HT(3) receptors on mitochondria influence mitochondrial function. *International Journal of Molecular Sciences,**24*(9). 10.3390/ijms24098301 10.3390/ijms24098301 PMC10179570 37176009

[5] Wrutniak-Cabello, C., Casas, F., & Cabello, G. (2001). Thyroid hormone action in mitochondria. *Journal of Molecular Endocrinology,**26*(1), 67–77. 10.1677/jme.0.0260067 11174855 10.1677/jme.0.0260067

[6] Reiter, R. J., Sharma, R., Rosales-Corral, S., de Campos Zuccari, D. A. P., & de Almeida Chuffa, L. G. (2022). Melatonin: A mitochondrial resident with a diverse skill set. *Life Sciences,**301*, 120612. 10.1016/j.lfs.2022.120612 35523285 10.1016/j.lfs.2022.120612

[7] Melhuish Beaupre, L. M., Brown, G. M., Goncalves, V. F., & Kennedy, J. L. (2021). Melatonin’s neuroprotective role in mitochondria and its potential as a biomarker in aging, cognition and psychiatric disorders. *Translational Psychiatry,**11*(1), 339. 10.1038/s41398-021-01464-x 34078880 10.1038/s41398-021-01464-x PMC8172874

[8] Hebert-Chatelain, E., Desprez, T., Serrat, R., Bellocchio, L., Soria-Gomez, E., Busquets-Garcia, A., et al. (2016). A cannabinoid link between mitochondria and memory. *Nature,**539*(7630), 555–559. 10.1038/nature20127 27828947 10.1038/nature20127

[9] West, E. E., Kunz, N., & Kemper, C. (2020). Complement and human T cell metabolism: Location, location, location. *Immunological Reviews,**295*(1), 68–81. 10.1111/imr.12852 32166778 10.1111/imr.12852 PMC7261501

[10] Arbore, G., & Kemper, C. (2016). A novel “complement-metabolism-inflammasome axis” as a key regulator of immune cell effector function. *European Journal of Immunology,**46*(7), 1563–1573. 10.1002/eji.201546131 27184294 10.1002/eji.201546131 PMC5025719

[11] Kolev, M., & Kemper, C. (2017). Keeping it all going-complement meets metabolism. *Frontiers in Immunology,**8*, 1. 10.3389/fimmu.2017.00001 28149297 10.3389/fimmu.2017.00001 PMC5241319

[12] Ishii, M., Beeson, G., Beeson, C., & Rohrer, B. (2021). Mitochondrial C3a receptor activation in oxidatively stressed epithelial cells reduces mitochondrial respiration and metabolism. *Frontiers in Immunology,**12*, 628062. 10.3389/fimmu.2021.628062 33746964 10.3389/fimmu.2021.628062 PMC7973370

[13] Ishii, M., & Rohrer, B. (2023). Anaphylatoxin C5a receptor signaling induces mitochondrial fusion and sensitizes retinal pigment epithelial cells to oxidative stress. *Biochimica et Biophysica Acta - General Subjects,**1867*(8), 130374. 10.1016/j.bbagen.2023.130374 37187450 10.1016/j.bbagen.2023.130374 PMC10330548

[14] Niyonzima, N., Rahman, J., Kunz, N., West, E. E., Freiwald, T., Desai, J. V., et al. (2021). Mitochondrial C5aR1 activity in macrophages controls IL-1beta production underlying sterile inflammation. *Science Immunology,**6*(66), eabf2489. 10.1126/sciimmunol.abf2489 34932384 10.1126/sciimmunol.abf2489 PMC8902698

[15] Sarti, A. C., Vultaggio-Poma, V., Falzoni, S., Missiroli, S., Giuliani, A. L., Boldrini, P., et al. (2021). Mitochondrial P2X7 receptor localization modulates energy metabolism enhancing physical performance. *Function (Oxf),**2*(2), zqab005. 10.1093/function/zqab005 35330818 10.1093/function/zqab005 PMC8788778

[16] Al Amir Dache, Z., & Thierry, A. R. (2023). Mitochondria-derived cell-to-cell communication. *Cell Reports,**42*(7), 112728. 10.1016/j.celrep.2023.112728 37440408 10.1016/j.celrep.2023.112728

[17] Al-Suhaimi, E., AlQuwaie, R., AlSaqabi, R., Winarni, D., Dewi, F. R. P., AlRubaish, A. A., et al. (2024). Hormonal orchestra: Mastering mitochondria’s role in health and disease. *Endocrine,**86*(3), 903–929. 10.1007/s12020-024-03967-1 39172335 10.1007/s12020-024-03967-1

[18] Schirrmacher, V. (2021). Less can be more: The hormesis theory of stress adaptation in the global biosphere and its implications. *Biomedicines,**9*(3). 10.3390/biomedicines9030293 10.3390/biomedicines9030293 PMC8000639 33805626

[19] Calabrese, E. J. (2018). Hormesis: Path and Progression to Significance. *International Journal of Molecular Sciences,**19*(10). 10.3390/ijms19102871 10.3390/ijms19102871 PMC6213774 30248927

[20] Picard, M., & Shirihai, O. S. (2022). Mitochondrial signal transduction. *Cell Metabolism,**34*(11), 1620–1653. 10.1016/j.cmet.2022.10.008 36323233 10.1016/j.cmet.2022.10.008 PMC9692202

[21] Klinge, C. M. (2020). Estrogenic control of mitochondrial function. *Redox Biology,**31*, 101435. 10.1016/j.redox.2020.101435 32001259 10.1016/j.redox.2020.101435 PMC7212490

[22] Ratajczak, M. Z., Bujko, K., Brzezniakiewicz-Janus, K., Ratajczak, J., & Kucia, M. (2024). Hematopoiesis revolves around the primordial evolutional rhythm of purinergic signaling and innate immunity - a journey to the developmental roots. *Stem Cell Reviews and Reports,**20*(3), 827–838. 10.1007/s12015-024-10692-9 38363476 10.1007/s12015-024-10692-9 PMC10984895

[23] Ratajczak, M. Z., Adamiak, M., Abdelbaset-Ismail, A., Bujko, K., Thapa, A., Chumak, V., et al. (2023). Intracellular complement (complosome) is expressed in hematopoietic stem/progenitor cells (HSPCs) and regulates cell trafficking, metabolism and proliferation in an intracrine Nlrp3 inflammasome-dependent manner. *Leukemia,**37*(6), 1401–1405. 10.1038/s41375-023-01894-0 37055506 10.1038/s41375-023-01894-0 PMC10244163

[24] Konopko, A., Lukomska, A., Kucia, M., & Ratajczak, M. Z. (2024). The Different Responsiveness of C3- and C5-deficient murine BM cells to oxidative stress explains why C3 deficiency, in contrast to C5 deficiency, correlates with better pharmacological mobilization and engraftment of hematopoietic cells. *Stem Cell Reviews and Reports,**22*, 43. 10.1007/s12015-024-10792-6 10.1007/s12015-024-10792-6 PMC11762589 39340736

[25] Ratajczak, M. Z., Bujko, K., Cymer, M., Thapa, A., Adamiak, M., Ratajczak, J., et al. (2020). The Nlrp3 inflammasome as a “rising star” in studies of normal and malignant hematopoiesis. *Leukemia,**34*(6), 1512–1523. 10.1038/s41375-020-0827-8 32313108 10.1038/s41375-020-0827-8 PMC7266743

[26] Bujko, K., Adamiak, M., Konopko, A., Chumak, V., Ratajczak, J., Brzezniakiewicz-Janus, K., et al. (2024). Defect in migration of HSPCs in Nox-2 deficient mice explained by impaired activation of Nlrp3 inflammasome and impaired formation of membrane lipid rafts. *Stem Cell Reviews and Reports,*. 10.1007/s12015-024-10775-7 39134888 10.1007/s12015-024-10775-7 PMC11762604

[27] Thapa, A., Ratajczak, J., Kucia, M., & Ratajczak, M. Z. (2023). External Liver-Derived complement and intrinsic present in hematopoietic stem/progenitor cells complosome modulate cell metabolism and response to stress. *Stem Cell Reviews and Reports,**19*(5), 1177–1184. 10.1007/s12015-023-10533-1 36976465 10.1007/s12015-023-10533-1 PMC10366307

[28] Burnstock, G. (2018). Purine and purinergic receptors. *Brain and Neuroscience Advances,**2*, 2398212818817494. 10.1177/2398212818817494 32166165 10.1177/2398212818817494 PMC7058212

[29] Ratajczak, M. Z., Thetchinamoorthy, K., Wierzbicka, D., Konopko, A., Ratajczak, J., & Kucia, M. (2025). Extracellular microvesicles/exosomes - magic bullets in horizontal transfer between cells of mitochondria and molecules regulating mitochondria activity. *Stem Cells (in press)*. 10.1093/stmcls/sxae086 10.1093/stmcls/sxae086 PMC11979747 39949038

